# Antibacterial Activity of Ethanol Extract of *Andrographis paniculata*

**DOI:** 10.4103/0250-474X.57294

**Published:** 2009

**Authors:** U. S. Mishra, A. Mishra, R. Kumari, P. N. Murthy, B. S. Naik

**Affiliations:** Royal College of Pharmacy and Health Sciences, Berhampur, Ganjam-760 002, India; 1 Seemanta Institute of Pharmaceutical Sciences, Jharpokharia, Mayurbhanj-757 086, India

**Keywords:** Antibacterial activity, *Andrographis paniculata*

## Abstract

In the present study the ethanol extract of the aerial part of *Andrographis paniculata* was prepared and evaluated for antimicrobial activity against eleven bacterial strains by determining minimum inhibitory concentration and zone of inhibition. Minimum inhibitory concentration values were compared with control and zone of inhibition values were compared with standard ciprofloxacin in concentration 100 and 200 μg/ml. The results revealed that, the ethanol extract is potent in inhibiting bacterial growth of both Gram-negative and Gram positive bacteria.

*Andrographis paniculata* (Acanthaceae) is an annual herb. It is found in Sri Lanka and throughout the plains of India specially Maharashtra, Karnataka, Uttar Pradesh, Tamilnadu, Orissa. Various medicinal properties like choleretic, antidiarrhoeal, immunostimulant and antiinflammatory have been attributed to this plant in the traditional system of Indian medicine[1–5]. Further reported activities are hepatoprotective, antimalarial, antihypertensive, antipyretic, antithrombotic and antidote for snake bites. The present investigation was undertaken to find out the antibacterial potentiality of the ethanol extract of the aerial part against some Gram positive and Gram negative bacteria.

The plants *Andrographis paniculata* were collected from the Similipal Biosphere Reserve, Mayurbhanj, Orissa and identified at Botanical Survey of India, Kolkata (Ref no.CNH/l-l (59)/2006/Tech.ll dated. 27.10.06). The aerial part of *Andrographis paniculata* were shade dried followed by drying in hot air oven for 30 min at low temperature, then it was powdered in a mechanical grinder. The dried powders were then used for extraction with suitable solvents.

The coarse dried powder of the aerial part (100 g) was subjected to extraction with 1200 ml ethanol for 48 h. The ethanol extract was collected, filtered and concentrated in vacuum under reduced pressure and dried in dessiccator. The yield was about 12.11% (w/w). The ethanol extract obtained was tested for the antimicrobial activity against eleven bacterial strains. These strains were clinical isolates from human beings and collected from the Department of Pharmaceutical Technology, Jadavpur University, Kolkata. All sub cultured microbes used were pure cultures preserved as slant agar culture at 4°. The concentrated ethanol extract was further subjected to preliminary phytochemical analysis[6,7].

The molten nutrient agar medium containing various concentrations of the extract (0, 10, 25, 50, 100 and 200 μg/ml) were poured and solidified onto sterile 100 mm Petri dishes to give sterile nutrient agar plates with varying dilutions of the extract. Then these plates were kept in a refrigerator (4°) for 24 h for uniform diffusion of the extract into the nutrient agar media. The plates were then dried at 37° for 2 h before spot inoculation[8]. One loopful (diameter 3 mm) of an overnight grown peptone water culture of each test organism was placed in Petri dish marked by checker board technique[9]. The spot inoculated plates were incubated at 37° for 24 h and the MIC values were obtained.

Ciprofloxacin was taken as a standard compound for comparing the results obtained with. Two sets of two dilutions (100 and 200 μg/ml) each of *Andrographis paniculata* aerial parts extract and ciprofloxacin (solvent: sterile distilled water) were prepared in sterile McCartney bottles. Sterile nutrient agar plates were prepared and incubated at 37° for 24 h to check for any sort of contamination. Two sterile filter paper discs (Whatman No. 1) of 6 mm diameter were soaked in two different dilutions of the crude extract and placed in appropriate position of the surface of the flooded plate, marked as quadrants at the back of the Petri dishes. The Petri dishes were incubated at 37° for 24 h and the diameter of zones of inhibition use measured in mm. Similar procedure was adopted for the pure ciprofloxacin and the corresponding zone diameters were compared accordingly[10].

The observations of the MIC study has been tabulated in Table 1 and it was found that the minimum inhibitory concentration of the ethanol extract was found to be varying between 10-200 μg/ml, with respect to most of the test bacteria. The MIC of ethanol extract for bacterial strain *E. coli K-12 row*, *Shigella sonnei-2*, *Salmonella typhi*-59, *V. cholera*-854, and *S. aureous*-ML-50 were found to be 100 μg/ml, for *V. cholera*-811, MIC was 50 μg/ml and for *S. aureous*-2737 and *B. licheniformis*-10341 were at 10 μg/ml. The result of ZOI of the extract and its comparison with standard antibiotic ciprofloxacin (100 μg/ml and 200 μg/ml) was recorded in Table 2. The antibacterial efficacy of extract of *Andrographis paniculata* was found to decrease in the following order against different tested bacterial strains- *Salmonella typhi*-59, *S. aureous*-2737, *V. alginolyteus*, *Sh. Boydii*-8, *V. cholera*-854, *E. coli* k-12 row, *B. licheniformis*-10341. From the results of MIC and zone of inhibition values and their competition to that of the standard ciprofloxacin, it evident that the ethanol extract is active against gram positive and gram negative bacteria.

**TABLE 1:** MIC OF ETHANOL EXTRACT OF *ANDROGRAPHIS PANICULATA* AGAINST DIFFERENT BACTERIA

Name of bacteria	Growth in nutrient agar containing different concentrations of extract (μg/ml)					
	
	0	10	25	50	100	200
*Escherichia coli* K 12 ROW	+	+	+	+	−	−
*Shigella sonnei* 2	+	+	+	+	−	−
*Salmonella typhi* 59	+	+	+	+	−	−
*Vibrio cholerae* 811	+	+	+	−	−	−
*Staphylococcus aureus* ML-59	+	−	−	−	−	−
*Shigella boydii* 8	+	+	+	+	−	−
*Bacillus licheniformis* 10341	+	−	−	−	−	−
*Salmonella typhimurium* NCTC 74	+	+	+	+	+	−
*Vibrio cholerae* 854	+	+	+	+	−	−
*Vibrio alginolyteus*	+	+	+	+	−	−
*Staphylococcus aureus* 29737	+	+	+	+	−	−

All determinations were done in triplicates.

‘0’ Control (without extract)

‘+’ Growth;

‘−’ No growth

**TABLE 2:** ZONES OF INHIBITION PRODUCED BY THE ETHANOL EXTRACT AND CIPROFLOXACIN

Name of bacteria	Ethanol extract		Ciprofloxacin	
		
	100 (μg/ml)	200 (μg/ml)	100 (μg/ml)	200 (μg/ml)
*Escherichia coli* K 12 ROW	6.3	7	10	19
*Staphylococcus aureus* 29737	6.3	9	22	25
*Staphylococcus aureus* ML 59	9	10	23	28
*Shigella boydii* 8	7	9	20	25
*Salmonella typhimurium* NCTC 74	6.2	6.8	23	29
*Shigella sonnei* 2	6.3	7	14	18
*Vibrio cholerae* 854	6.3	8	22	28
*Vibrio cholerae* 811	10	13	15	20
*Salmonella typhi* 59	13	14	27	35
*Vibrio alginolyteus*	7	10	26	29
*Bacillus licheniformis* 10341	6.2	7	17	27

Zone of inhibition, including the diameter of the filter paper disc (6 mm); mean value of three independent experiments; ciprofloxacin was used as positive control.

The compounds responsible for this antibacterial activity have not been investigated. However, preliminary phytochemical analysis of the ethanol extract revealed the presence of carbohydrates, tannins, flavonoids and saponins[6,7]. The antibacterial properties of the plant may be attributed to the individual or combined effect of the above mentioned chemical groups. The findings of the present investigation offer a scientific support to the ethnomedicinal use of the plant by the traditional healers.
